# Surface functionalization of electrospun scaffolds using recombinant human decorin attracts circulating endothelial progenitor cells

**DOI:** 10.1038/s41598-017-18382-y

**Published:** 2018-01-08

**Authors:** Svenja Hinderer, Katrin Sudrow, Maria Schneider, Monika Holeiter, Shannon Lee Layland, Martina Seifert, Katja Schenke-Layland

**Affiliations:** 1 Department of Cell and Tissue Engineering, Fraunhofer-Institute for Interfacial Engineering and Biotechnology (IGB), 70569 Stuttgart, Germany; 2 Department of Women´s Health, Research Institute for Women’s Health, Eberhard-Karls-University Tübingen, 72076 Tübingen, Germany; 3 Institute of Medical Immunology and Berlin-Brandenburg Center for Regenerative Therapies (BCRT), Charité-Universitätsmedizin Berlin, corporate member of Freie Universität Berlin, Humboldt-Universität zu Berlin, and Berlin Institute of Health, 13353 Berlin, Germany; 4 Department of Medicine/Cardiology, Cardiovascular Research Laboratories, David Geffen School of Medicine at UCLA, Los Angeles, CA USA

## Abstract

Decorin (DCN) is an important small leucine-rich proteoglycan present in the extracellular matrix (ECM) of many organs and tissues. Endothelial progenitor cells (EPCs) are able to interact with the surrounding ECM and bind to molecules such as DCN. Here, we recombinantly produced full-length human DCN under good laboratory practice (GLP) conditions, and after detailed immunological characterization, we investigated its potential to attract murine and human EPCs (mEPCs and hECFCs). Electrospun polymeric scaffolds were coated with DCN or stromal cell-derived factor-1 (SDF-1α) and were then dynamically cultured with both cell types. Cell viability was assessed via imaging flow cytometry. The number of captured cells was counted and compared with the non-coated controls. To characterize cell-scaffold interactions, immunofluorescence staining and scanning electron microscopy analyses were performed. We identified that DCN reduced T cell responses and attracted innate immune cells, which are responsible for ECM remodeling. A significantly higher number of EPCs attached on DCN- and SDF-1α-coated scaffolds, when compared with the uncoated controls. Interestingly, DCN showed a higher attractant effect on hECFCs than SDF-1α. Here, we successfully demonstrated DCN as promising EPC-attracting coating, which is particularily interesting when aiming to generate off-the-shelf biomaterials with the potential of *in vivo* cell seeding.

## Introduction

Cells in a tissue are surrounded by a highly heterogenic and complex network of structural and functional molecules - the extracellular matrix (ECM). The ECM serves as a scaffold for cells, but more important, it provides biomechanical and biochemical cues, which are required for cellular responses such as migration, proliferation and differentiation^1^. There exist various ECM macromolecules such as fibrillar proteins, including collagens and elastic fibers, fibronectin and laminins, as well as functional components like water- and growth factor-binding proteoglycans and glycosaminoglycans^1,2^. Decorin (DCN) for example, is a small leucine-rich proteoglycan consisting of a core protein, which is covalently linked to one glycosaminoglycan chain^3^. It has been reported, that DCN plays a significant role in collagen fibrillogenesis^3,4^ and skeletal muscle differentiation^5^. Furthermore, DCN is highly expressed in maturing and adult heart valves^6^, and enables tracheal cell culture while possessing an immunomodulatory capacity^7^. Growth factors such as transforming growth factor beta (TGF-β) or insulin-like growth factor-1 (IGF-1) are able to bind to DCN^3,8^. In addition, the vascular endothelial growth factor receptor-2 (VEGFR2), which is expressed by endothelial progenitor cells (EPCs), has a DCN affinity^9^.

In a previous study, we developed an electrospun scaffold, composed of poly (ethylene glycol) dimethacrylate and poly (L-lactide) (PEGdma-PLA), which was based on the histoarchitecture and the biomechanical properties of a native heart valve leaflet^10^. Our overall goal is to generate a cell-free, off-the-shelf heart valve material that has the potential to attract EPCs from the circulation or the surrounding tissue after implantation and potentially supports tissue growth. The production of cell-free implants with the potential of *in vivo* cell seeding is less expensive and time consuming compared to pre-seeded tissue-engineered products (Advanced Therapy Medicinal Products - ATMPs)^11^. Previously, cell infiltration from the surrounding tissue has been enabled by modifying the topography^12^ or by introducing proteins^13^, polysaccharides^14^, RGD-sequences and chemokines^15,16^. Another successful approach is to recruit progenitor cells from circulating blood by providing chemokines such as stromal cell-derived factor-1 alpha (SDF-1α). SDF-1α is a well-known chemo-attractant, binding to the CXC receptor 4 (CXCR4) of EPCs^17,18^. SDF-1α not only promotes cell adhesion, but is also involved in endothelial cell differentiation^17^. It plays a crucial role in vascular remodeling^19^ and furthermore, it has been demonstrated that SDF-1α recruits EPCs to the ischemic heart muscle and induces vasculogenisis^15^.

In this study, we aimed to produce preclinical good laboratory practice (GLP)-compliant full-length human recombinant DCN using Chinese hamster ovary (CHO) cells and to analyze its potential effect on innate and adaptive human immune responses. Furthermore, we assessed the attraction potential of DCN-coated electrospun polymeric scaffolds to circulating EPCs under dynamic cell culture conditions, and compared it with the *in vitro* EPC attraction capacity of the chemokine SDF-1α.

## Results

### Production of human recombinant DCN in CHO cells

**Figure 1:**
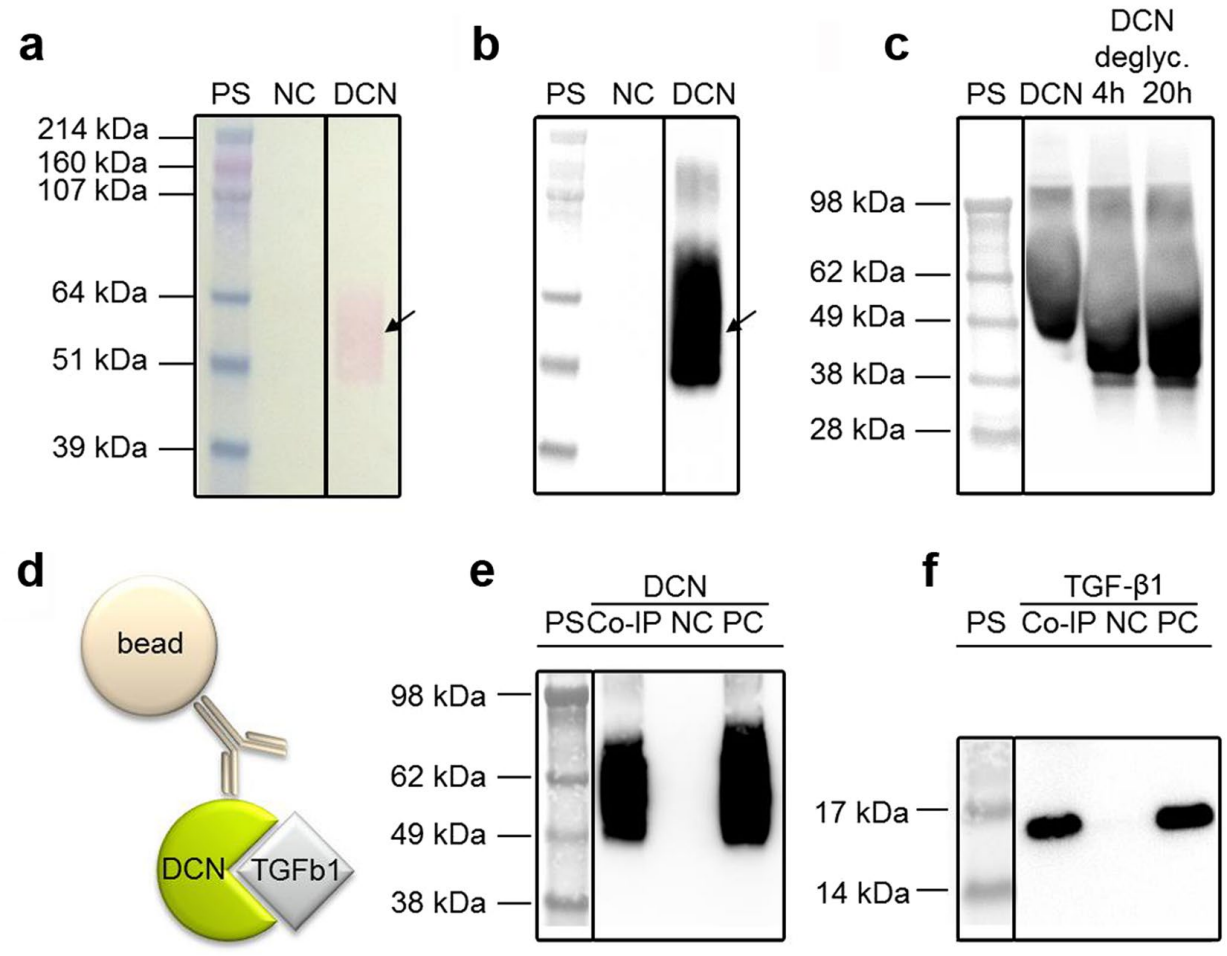
Characterization of recombinant human DCN. **(a)** Ponceau-Red staining of a nitrocellulose membrane containing an IMAC purification of suspension media with 1% dialyzed FBS (negative control, NC), an IMAC purification of DCN-containing cell culture media with originally 1% dialyzed FBS (DCN), and the HiMark Pre-stained protein standard (PS). **(b)** The same membrane after discoloring and specific immunodetection of DCN. **(c)** Specific immunodetection of untreated DCN, deglycosylated DCN (DCN deglyc., 4 h and 20 h) and the SeeBlue® Plus2 Pre-stained protein standard (PS). **(d)** Schematic of a Co-IP for the protein interaction between DCN and TGF-β1. **(e,f)** Specific immunodetection of human DCN **(e)** or TGF-β1 from human platelets **(f)** show the Co-IP eluate (Co-IP), an unspecific background control without the DCN antibody (NC), the positive control (PC; 250 ng DCN or 100 ng TGF-β1) and the SeeBlue® Plus2 Pre-stained protein standard (PS). All blots are cropped, indicated by the black boxes. Full-length blots are presented in Supplementary Figure 7.

The expression plasmid was designed to have the complete DCN expression cassette in close proximity to the DHFR cassette, which increased the chance that these protein cassettes were co-amplified. Genomic co-amplification of the DHFR and DCN gene resulted in a significantly increased DCN production (Supplementary Fig. S1) with concentrations of up to 42.8 µg/mL DCN in the production media after three MTX selection rounds. The production clones that provided the highest yields as determined by DCN ELISA were adapted to suspension culture and serum depletion, followed by production up-scaling and protein purification using fast protein liq uid chromatography (FPLC)-controlled immobilized metal affinity chromatography (IMAC). With this purification method, based on the natural affinity of the amino acid histidine to immobilized nickel ions on an affinity chromatography column as well as an additional protein eluate desalting and concentration step, 78% of the initial DCN content were retained. The identity of the DCN was determined using SDS PAGE and Western blot (Fig. 1a,b). Deglycosylation was successful, indicated by the DCN band shift to a lower protein size (Fig. 1c). To proof functionality of the human recombinant DCN regarding TGF-β1 binding, Co-IP studies were performed (Fig. 1d–f). By using specific antibodies on the blotted membranes, the interaction between DCN and TGF-β1 was visualized (Fig. 1d,f). Specific immunodetection showed that the Co-IP eluate does not only contain decorin, but also TGF-β1, which is not present in the negative control eluate. Therefore, the Co-IP shows the specific binding of TGF-β1 by decorin.

### Immune responses induced by human recombinant DCN

**Figure 2:**
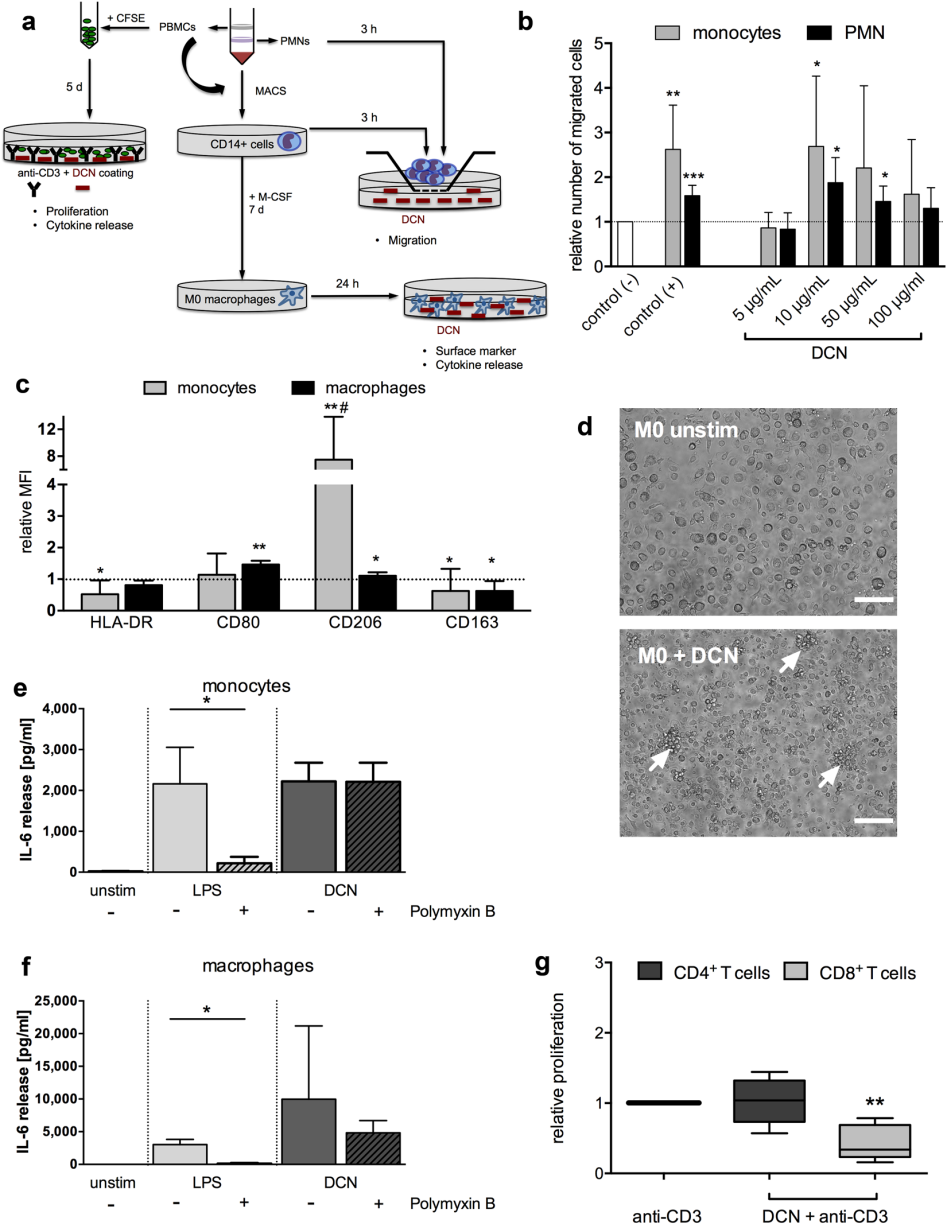
Evaluation of human immune responses induced by human recombinant DCN *in vitro*. **(a)** The experimental design for the immunological characterization of DCN including: isolation of human PBMCs and labeling with CFSE for immune cell proliferation analysis, isolation of PMNs, CD14+ monocytes and M0 type macrophages for testing migration and changes in surface marker expression and cytokine release by flow cytometry and ELISA. **(b)** Migration of CD14+ monocytes and PMNs towards different DCN concentrations or 10% human serum (positive control) after 3 hours. *P < 0.05 **p < 0.01 ***p < 0.001 relative to the control (−) with one-way ANOVA and Dunnet’s post test; n = 3 with 9 single values. **(c)** Classical cell surface markers were determined after co-culture of CD14+ monocytes for 7 days or M0 type macrophages for 24 hours with 50 µg/mL DCN, and the relative mean fluorescence intensities (MFI) ± SD are shown in comparison with the untreated control (set as 1). Data were analyzed for each marker by a Kolmogorov-Smirnov-test; *p < 0.05, **p < 0.01 compared to control or two-way- ANOVA with Sidak’s post test; #p < 0.001 comparing both cell types; n = 5. **(d)** Representative images of unstimulated M0 type macrophages (M0 unstim) and DCN-stimulated macrophages (M0 + DCN) with cell cluster formation (white arrows). Scale bars equal 100 µm. Release of IL-6 in co-cultures of monocytes **(e)** and M0 macrophages **(f)** with either DCN (50 µg/mL) or lipopolysaccharide (LPS; 10 µg/mL) alone, or after pre-incubation with Polymyxin B (50 µg/mL) compared to unstimulated (unstim) cultures. Data were analyzed by Mann-Whitney U test; *p < 0.05; n = 5. **(g)** Proliferation of T cells after a 5 day-co-culture of human PBMCs with either low-dose anti-CD3 alone or DCN combined with anti-CD3 (DCN + anti-CD3) was tested in a CFSE-based assay and measured by flow cytometry. The relative proliferation of CD4+ and CD8+ T cells relative to the anti-CD3-stimulated control (set as 1) is shown. Data were analyzed by Kruskal-Wallis test with Dunn’s post test; **p < 0.01; n = 6.

Based on the well-known interaction of DCN from other species with human immune cells^3,20,21^, we evaluated human immune responses induced by the generated human recombinant DCN. Human PBMC, PMNs, purified CD14^+^ monocytes and GM-CSF-generated M0 type macrophages were analyzed in appropriate *in vitro* test settings shown schematically in Fig. 2a for induction of: (1) immune cell proliferation, (2) cytokine secretion, (3) migration, as well as (4) differentiation or polarization. First, purified CD14^+^ human monocytes and PMNs were seeded on the membrane of a chemotaxis system with a DCN concentration gradient and the number of migrated cells was detected after 3 hours. The relative number of migrated cells was calculated by setting the spontaneous migration value without the presence of DCN (negative control) equal to 1. DCN induced a significant, dose-dependent migration of human monocytes and PMNs when compared with the negative control at concentrations of 10 and 50 µg/mL, respectively (Fig. 2b). Next, we tested whether the presence of human DCN influences surface marker expression on co-cultured monocytes or macrophages. Therefore, either CD14^+^ monocytes or M0 type macrophages, generated by 7-day stimulation of CD14^+^ monocytes with GM-CSF, were incubated with 50 µg/mL DCN and screened for changes in their cell morphology and their surface marker pattern. The results of flow cytometric analysis, performed after gating on all single and viable cells, are shown for macrophages in Supplementary Fig. S2. After 7 days of culture in the presence of DCN, monocytes significantly up-regulated CD206 and slightly down-regulated HLA-DR and CD163 (Fig. 2c). In contrast, for M0 type macrophages cultured with DCN, we could detect a significant increase of the co-stimulatory molecule CD80, but no changes in HLA-DR, which is another activation and M1 type marker (Fig. 2c). In addition, small changes were also detected for CD206 (upregulated) and CD163 (downregulated) (Fig. 2c). Morphologically, M0 type macrophages induced the formation of small cell clusters in cultures with DCN (Fig. 2d).

When analyzing the cytokine secretion in co-cultures of CD14^+^ monocytes and M0 type macrophages with DCN, we detected elevated levels for TNFα and IL-10 (Supplementary Fig. S3) when compared with the unstimulated control cultures. For both cytokines, the release from monocytes could be partially inhibited by the addition of Polymyxin B, but this was not true for macrophages (Supplementary Fig. S3). Interestingly, Polymyxin B could not inhibit IL-6 secretion from either cell subset in contrast to the LPS-stimulated controls (Fig. 2e,f).

Next, we asked if human DCN in its full recombinant form is also able to influence adaptive immune mechanisms and tested the ability to modulate an anti-CD3-triggered T cell proliferation as a second trigger for memory cells in a CFSE-based assay (Fig. 2a). After gating on single living cells, we analyzed the CFSE-signal for the subsets of both CD3^+^CD4^+^ and CD3^+^CD8^+^ T cells (Supplementary Fig. S2). Unexpectedly, we identified that DCN was able to dampen the anti-CD3 triggered response exclusively for CD8^+^ T cells (Fig. 2g), but not for the CD4^+^ T cell subset. DCN also significantly reduced the secretion of IL-10 in those PBMC co-cultures after 5 days, but not of TNFα and IFNγ (Supplementary Fig. S4).

### Viability assessment of mEPCs and hECFCs cultured on protein-coated scaffolds

**Figure 3:**
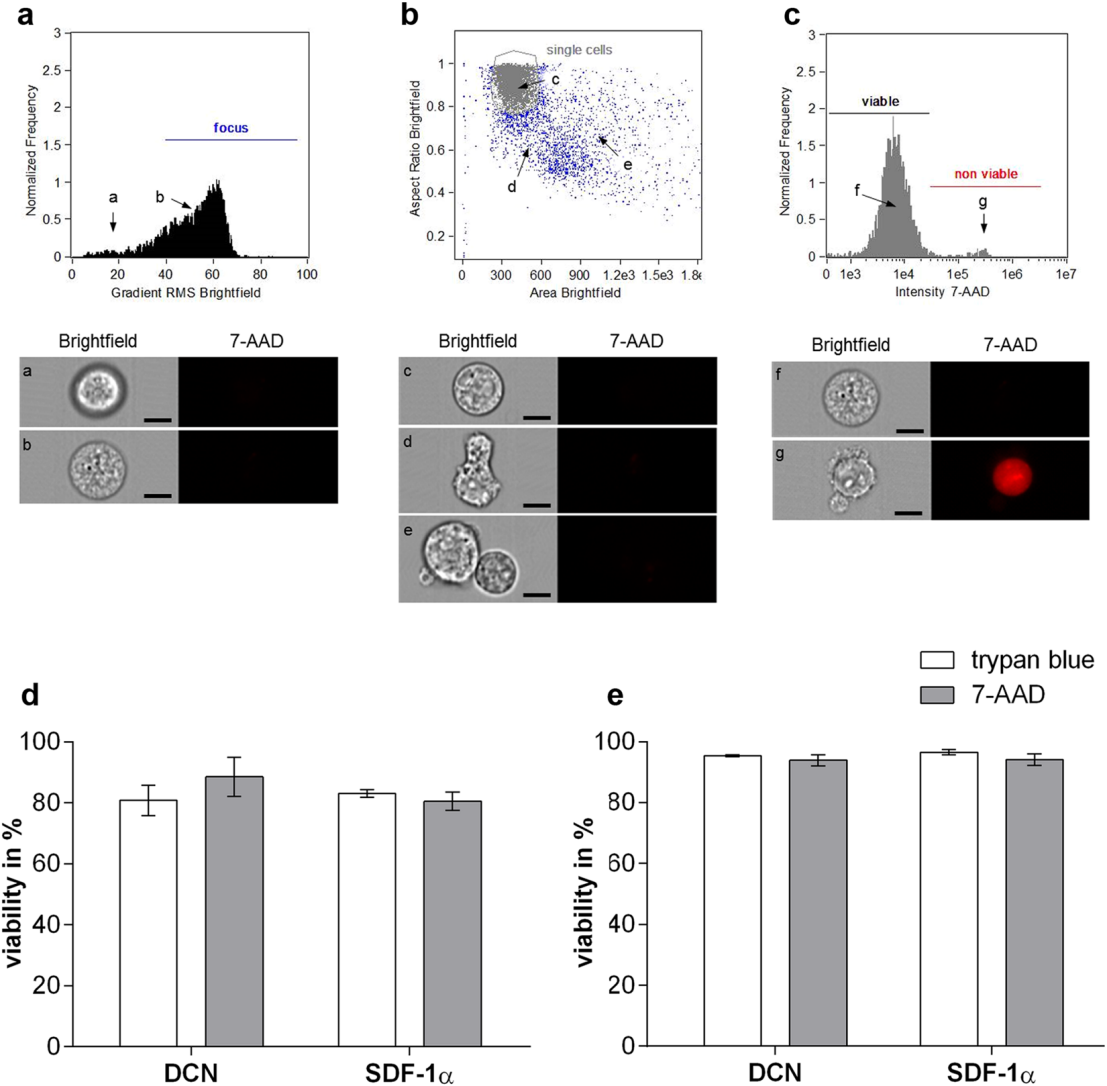
Imaging flow cytometry analysis. (**a**–**c**) Settings for analysis. Histograms with the corresponding bright field and fluorescence images of single cells. Dead cells are stained red employing 7-AAD. **(a)** Histogram shows cells out of focus (**a**) and in focus (**b**). Only cells in focus were used for further analysis. **(b)** In a next step, single cells were selected (**c**). 7-AAD negative debris (**d**) and cell agglomerates (**e**) were excluded from further analysis. **(c)** The Histogram shows viable ((**f**) 7-AAD negative) and non viable ((**g**) 7-AAD positive) cells. Scale bars equal 10 µm. Cell viability of **(d)** mEPCs and **(e)** hECFCs on protein-coated scaffolds using trypan blue or imaging flow cytometry with 7-AAD (n = 3).

Here, we used the chemokine SDF-1α as a positive control. This molecule is well known for recruiting CXCR4 positive cells and has already been used in several studies for EPC immobilization^22–24^. After successful and stable coating of the PEGdma-PLA scaffolds with DCN or SDF-1α (Supplementary Fig. S6), potential cytotoxic effects were investigated utilizing mEPCs and hECFCs. Cell viability was assessed and quantified using imaging flow cytometry employing 7-AAD staining (Fig. 3). The results of these experiments were compared with a standard trypan blue stain, revealing a mEPC viability of more than 80% (Fig. 3d), and a hECFC viability of almost 100% (Fig. 3e) when cultured on DCN-coated and SDF-1α-coated scaffolds.

### Cell capture evaluation in a dynamic culture system

**Figure 4:**
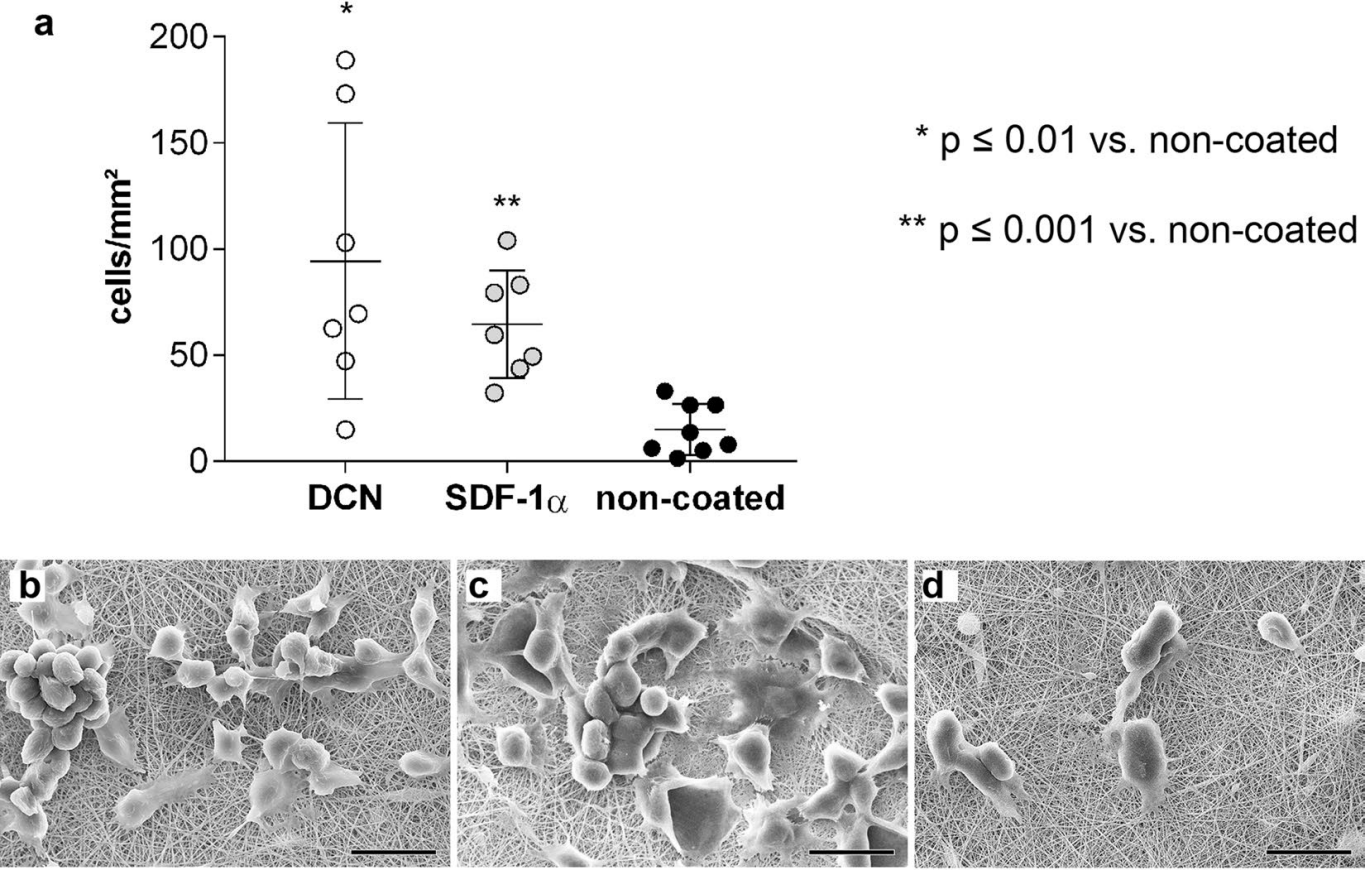
Attraction of mEPCs to coated and non-coated scaffolds. **(a)** Number of attached cells per mm^2^ on coated and non-coated electrospun PEGdma-PLA scaffolds. Corresponding SEM images of attached mEPCs on scaffolds coated with **(b)** DCN, **(c)** SDF-1α, and **(d)** non-coated scaffolds (n = 8 each). Scale bars equal 20 µm.

**Figure 5:**
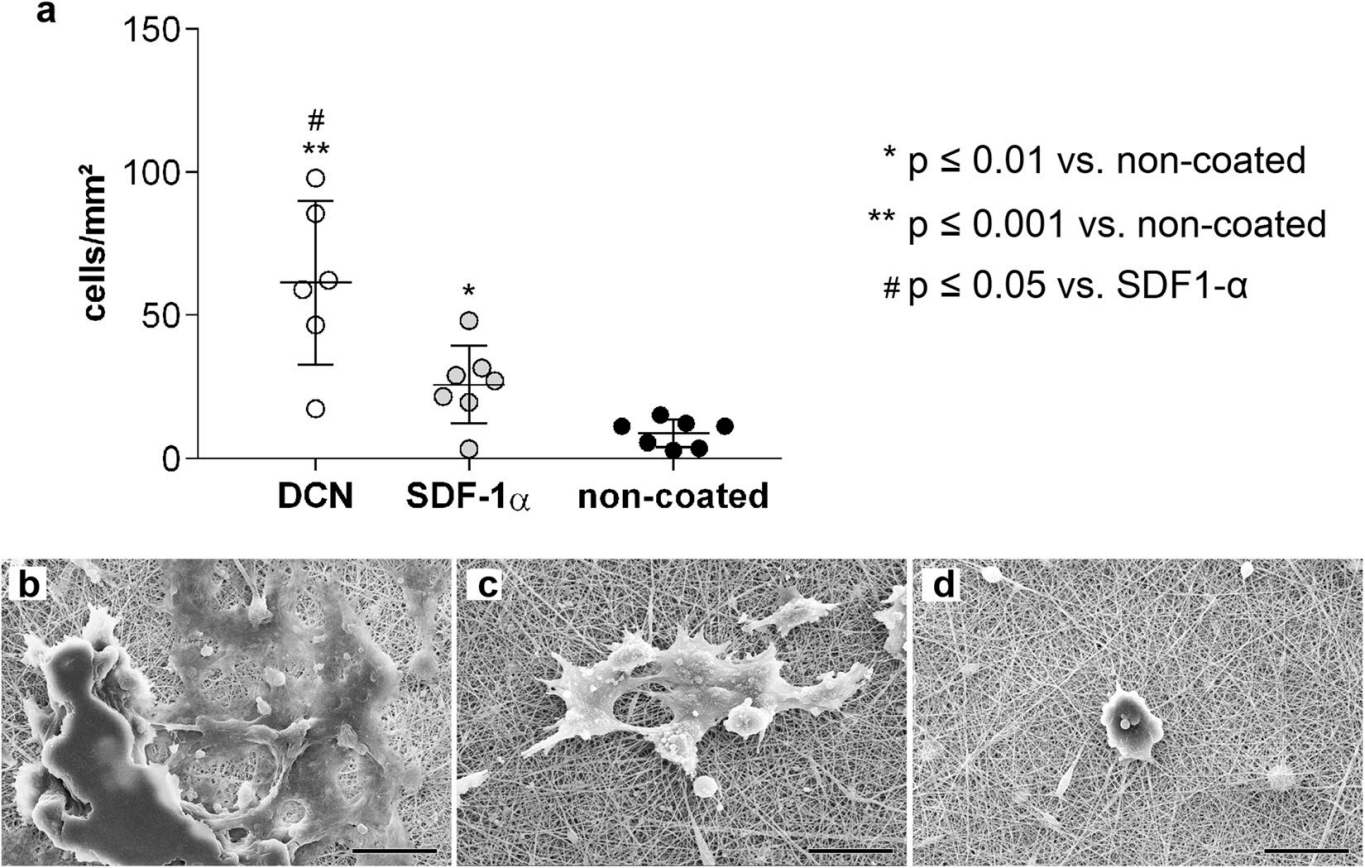
HECFCs capture experiments under dynamic conditions. **(a)** Number of attached cells per mm^2^ on coated and non-coated electrospun PEGdma-PLA scaffolds. Corresponding SEM images of attached hECFCs on scaffolds coated with **(b)** DCN, **(c)** SDF-1α, and **(d)** non-coated scaffolds (n = 8 each). Scale bars equal 20 µm.

In order to detect the capacity of different scaffold coatings to attract circulating murine and human progenitor cells, we incubated the scaffolds in a cell suspension on a rotary shaker under continuous agitation. For cell number assessment, the attached cells on the scaffolds were labelled with DAPI and counted (Figs 4a and 5a). In addition, cell morphology and distribution was evaluated using SEM (Fig. 4b–d; Fig. 5b–d).

We identified, that after 48 hours of dynamic culture, a significantly higher number of mEPCs was detected on both the DCN-coated (83 cells/mm^2^; 95% CI 34–154 cells/mm^2^; p ≤ 0.01) and SDF-1α-coated (65 cells/mm^2^; 95% CI 41–84 cells/mm^2^; p ≤ 0.001) scaffolds when compared with the non-coated PEGdma-PLA scaffolds (15 cells/mm^2^; 95% CI 5–25 cells/mm^2^) (Fig. 4a). The level of significance was higher for the mEPCs cultured on SDF-1α-coated scaffolds when compared with DCN-coated scaffolds, although the average number of attached cells was higher on the DCN-coated scaffolds. SEM analyses revealed that the mEPCs displayed a less spread morphology on the DCN-coated scaffolds when compared with the SDF-1α-coated scaffolds (Fig. 4b versus c); however, no difference in size was detected. Only a few single cells were determined on the non-coated PEGdma-PLA scaffolds (Fig. 4D).

Attempting to transfer this dynamic culture system to hECFCs, we found that these cells died when being exposed more than 24 hours to suspension culture conditions. Therefore, all analyses utilizing hECFCs were then performed after 24 hours of culture, instead of 48 hours. We identified that the highest number of hECFCs adhered to the DCN-coated scaffolds (DCN: 61 cells/mm^2^; 95% CI 31–91 cells/mm^2^ versus non-coated control: 9 cells/mm^2^; 95% CI 4–13 cells/mm^2^; p ≤ 0.001), although significance was also seen utilizing SDF-1α coating when compared with the non-coated controls (SDF-1α: 26 cells/mm^2^; 95% CI 13–38 cells/mm^2^; p ≤ 0.01) (Fig. 5). Interestingly, and in contrast to the experiments performed using murine cells, where the same number of cells attached to DCN- and SDF-1α-coated scaffolds, a significantly higher number of hECFCs were attracted and attached to the DCN-coated scaffolds (p ≤ 0.05 for DCN versus SDF-1α). SEM image analyses confirmed this data, showing that the two coatings impacted cell morphologies. In detail, DCN- and SDF-1α-coated scaffolds were covered with spreading hECFCs (Fig. 5b and c). A clear separation of single cells and therefore a morphological differentiation was not possible, since the edges of the cells overlapped. In contrast, almost no hECFCs adhered to the non-coated PEGdma-PLA scaffolds, and the few cells that did attach, were rounded (Fig. 5d).

### Cell-scaffold interactions

**Figure 6:**
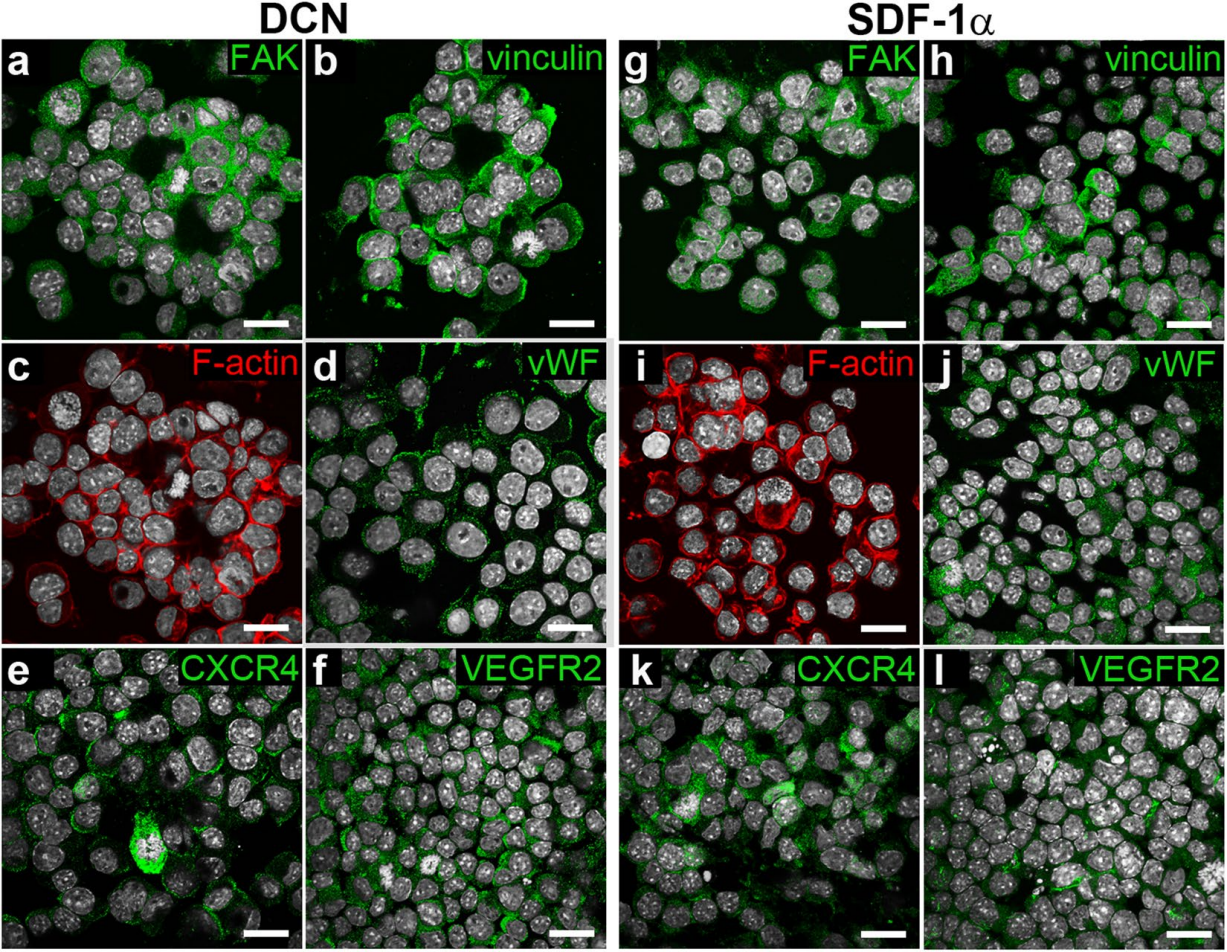
Immunofluorescence staining of adhered mEPCs on PEGdma-PLA scaffolds coated with DCN **(a**–**f)** or SDF-1α **(g**–**l)** after 48 hours of dynamic culture. Cell nuclei are shown in white (DAPI). Scale bars equal 20 µm.

**Figure 7:**
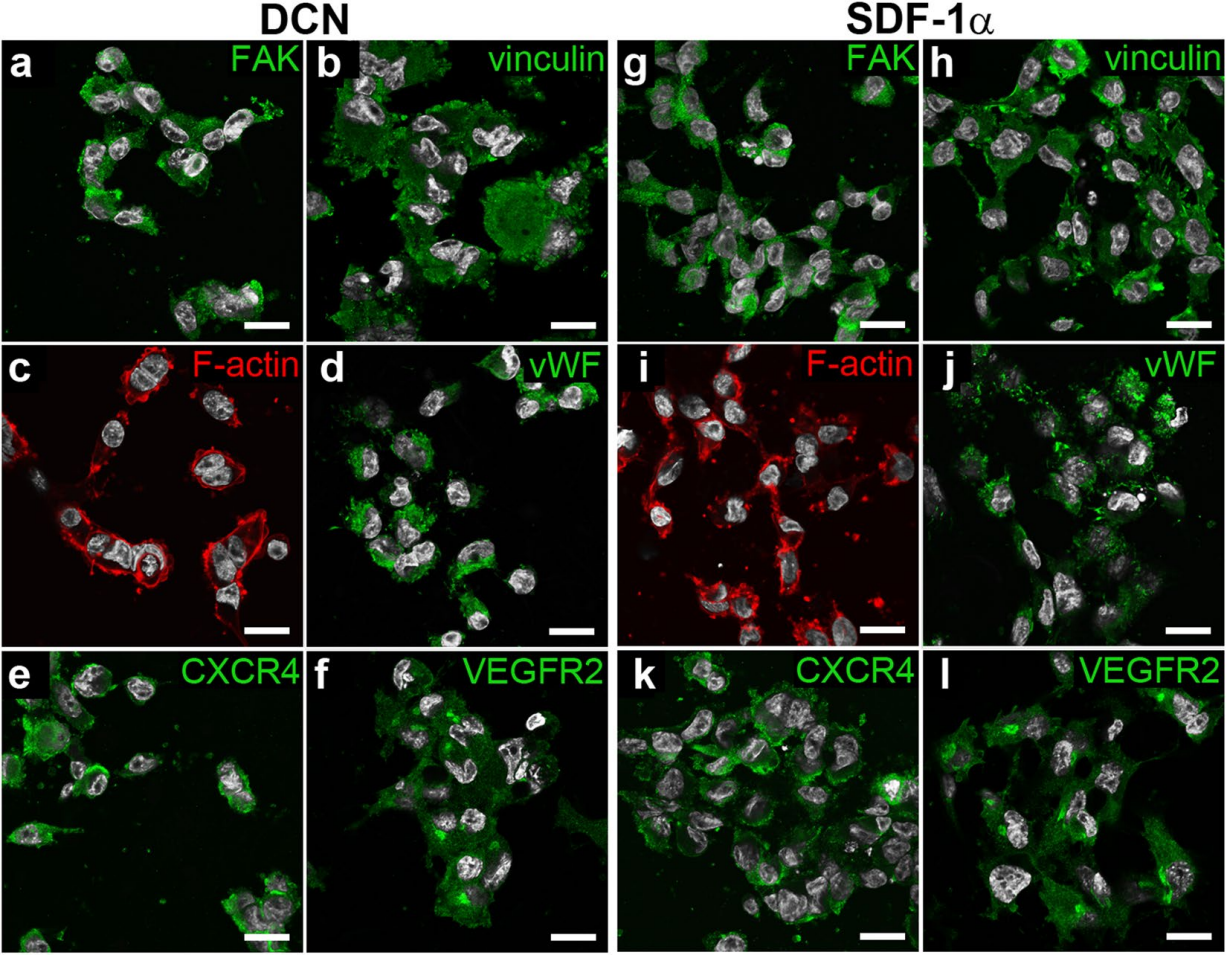
Immunofluorescence staining of adhered hECFC on PEGdma-PLA scaffolds coated with DCN **(a**–**f)** or SDF-1α **(g**–**l)** after 24 hours of dynamic culture. Cell nuclei are displayed in white (DAPI). Scale bars equal 20 µm.

In order to visualize cell-material interaction behavior, we performed antibody staining of the attached mEPCs after 48 hours (Fig. 6), and hECFCs after 24 hours (Fig. 7) of culture on the DCN- and SDF-1α-coated electrospun PEGdma-PLA scaffolds. The cell adhesion markers vinculin and focal adhesion kinase (FAK) were equally expressed by both cell types on DCN- and SDF-1α-coated scaffolds. Furthermore, the endothelial cell marker vWF was visualizable on the majority of murine and human progenitor cells on either scaffold. Also, the receptors CXCR4 and VEGFR2, which are important for the cell binding to DCN and SDF-1α, were expressed under all conditions.

## Discussion

The ECM and its components provide fundamental cell-instructive cues. To utilize ECM molecules either as a biomaterial or to include them as a surface modification of an implant potentially enables the generation of fully functional and biocompatible scaffolds^25^.

In the present study, we analyzed whether the coating of a polymer-based scaffold with the ECM protein DCN could attract EPCs and allow the cells to attach to the engineered substrate. For this purpose, we described a method to produce full-length human recombinant DCN using CHO cells under GLP conditions, which, to our knowledge, is so far not available and would enable future applications in humans. In our immunological compatibility studies, we showed effects that were so far described only for human DCN fragments or for DCN of other species. As previously known for DCN from bovine ligaments^7^ we also saw enhanced secretion of TNFα, but in addition also of IL-6 and IL-10 in co-cultures with human CD14^+^ monocytes. Triggering of TNFα by binding of DCN to Toll-like receptor (TLR) 2/4 and followed by activating NFkB was described by others before^26,27^. After treatment with Polymyxin B, which is a potent reagent to neutralize LPS, we found no inhibition of the IL-6 secretion by monocytes and macrophages in contrast to the LPS-treated cultures arguing for DCN-specific responses, excluding effects mediated by LPS contamination. However, besides its known pro-inflammatory action, IL-6 can also have anti-inflammatory properties^28^ by classical signaling via the IL-6 receptor expressed on some cell types including macrophages^29^ and might therefore support regenerative processes. In addition, the anti-inflammatory cytokine IL-10 is also triggered via DCN binding to TLR2/4^27^.

Aiming for usage of DCN as a component for the bio-functionalization of tissue-engineered scaffolds in skin, musculoskeletal or cardiovascular applications, its impact on migration of monocytes into the tissue and on the activation or even polarization of macrophages within the tissue is of great importance. So far, no information was described regarding these features for human DCN. Here, we identified for the first time that DCN serves as a chemotactic stimulus for either monocytes or PMNs (Fig. 2b). We also showed that culturing monocytes in the presence of DCN changes some of their surface markers, especially upregulating CD206. The induction of this well-known mannose receptor might be a sign of differentiation into macrophages and the enhanced capacity for antigen uptake and presentation^30,31^. DCN might act by binding via its GAG part to the mannose receptor, inducing CD206 upregulation on monocytes as known for the C-type Lectin Receptors in general^32^. When analyzing the influence of human DCN on the macrophage polarization process in co-culture studies with *in vitro*-generated M0 type macrophages, we found up-regulation of some surface molecules, like the co-stimulation marker CD80 (Fig. 2c). In general, tissue macrophages might be triggered to polarize into two directions depending on the tissue milieu: the more pro-inflammatory M1 type and the more regenerative M2 type as described by several other groups^33–35^. The enhanced CD80 expression level is similar to the characteristics of *in vitro* generated M1 type macrophages, as shown in the marker profile of control cell cultures (Supplementary Fig. S5). However, the typical HLA-DR increase on M1 macrophages was not induced by DCN. Macrophages in DCN co-cultures also showed a slight up-regulation of the marker CD206, which is assumed to be an M2 type marker, although there are still discussions about the reliability of some surface markers for characterizing the M1 versus M2 types^36,37^. The cytokine secretion profile of macrophage/DCN co-cultures showed similarity with that of M1 type macrophages with high secretion levels for TNFα, but also IL-6 and IL-10 (Supplementary Fig. S4). We might speculate, that similar to the mouse system, the enhanced M1 type cytokine levels are mediated by trapping the endogenously produced TGF-β by DCN^20^.

Our analysis of induced T cell proliferation showed a suppressive effect on exclusively CD8^+^ cytotoxic T cells (Fig. 2g), which was in accordance to our own experimental data using bovine DCN^7^. Inhibition of CD8^+^ T cell proliferation might be caused by the significant inhibition of IL-10 secretion we observed in our co-culture setting (Supplementary Fig. S4). Besides the well-known anti-inflammatory effects of IL-10 that occur by deactivating monocytes/macrophages and dendritic cells^38^, it is able to stimulate the proliferation of CD8^+^ T cells^39^. The inhibitory effect of DCN on IL-10 generation is mediated via the enhanced expression of programmed cell death (PDCD) 4, a translational suppressor of IL-10^21,27^. That DCN could act as a suppressor of cell proliferation was already shown for other cell types like endothelial cells and macrophages^40,41^.

We further aimed to modify the surface of an electrospun synthetic PEGdma-PLA scaffold with the here produced DCN in order to assess its capacity to attract circulating progenitor cells. As a control substance, we used the chemokine SDF-1α in our study since it had been previously shown that SDF-1α enhances the homing of progenitor cells from the bone marrow^24^, and it is currently clinically tested for its potential to induce tissue repair through recruitment of stem and progenitor cells^22,23^, although the benefit of this strategy is controversially discussed in the clinical field^22^. Although PEG is known for its anti-fouling properties^42,43^, it was possible to physically adsorb the proteins to an electrospun PEGdma-PLA hybrid.

In our study, both molecules, DCN and SDF-1α, attracted mEPC and hECFC in the dynamic *in vitro* cultures and significantly supported cell attachment when compared with the performance of non-coated PEGdma-PLA scaffolds. Progenitor cell recruitment and migration using SDF-1α has already been investigated for vascular grafts^11^, indicating that this is a highly interesting candidate for implant functionalization. However, the lifetime of SDF-1α in blood is according to Misra *et al*. only around 25.8 minutes^44^, which represents a huge limitation. In order to achieve a prolonged effect of SDF-1α, protection strategies are necessary. Lee *et al*. generated a coacervate composed of poly(ethylene argininylaspartate diglyeride), heparin and SDF-1α, which was employed as a coating for a vascular graft. Due to the polymer, the SDF-1α was protected from the surrounding medium^11^. Another approach is to directly electrospin SDF-1α together with the PEGdma-PLA. We have previously reported on a method to electrospin proteins without impacting their function^7^. In this study, the molecule was present on the surface and within the biodegradable fiber. Harnessing this approach, a protein-release by normal degradation could potentially ensure the availability of the functional SDF-1α over a longer period of time. Also other substances such as the non-specific RGD-sequenz, the chemokine CXCL-1^16^, or the monocyte chemotactic protein 3 (MCP3)^45^ have been described to recruit endothelial cells or enable cell homing.

Interestingly, using DCN as coating, we saw a significantly higher number of hECFCs attached on the scaffolds, when compared with the SDF-1α coating or non-coated scaffolds, indicating a potential benefit of the human ECM protein when using human cells. Considering the binding of cells to DCN via the VEGFR-2^9^, the application of DCN as an implant coating needs to be carefully assessed. Besides all the positive effects of DCN such as lowering T cell responses^7^, inducing tumor growth inhibition^21,46,47^, enabling cell differentiation^48^ or contributing to collagen fibrillogenesis^9^, there are also reports on DCN-induced signaling pathways downstream the VEGFR2, causing autophagy^49,50^. However, in these studies only the protein core of DCN was employed and investigated. In contrast, here, we use a human recombinant full-length DCN with a His-tag for scaffold modification. The cell attracting properties of this material are highly interesting when aiming for a smart material, where specific cells are able to adhere after implantation. This enables the production of functional off-the-shelf implants. The protein can either be directly electrospun into the scaffold to provide a ready-to-use product, or applied as a coating right before implantation. Compared to other cell recruiting chemokines, DCN is quite versatile since it also binds important growth factors and is involved in matrix assembly^9^. Especially in the field of heart valve engineering, DCN seems to be an extremely potent molecule, since it is highly expressed during heart valve development^6^.

We further investigated cell-material interactions using immunofluorescence staining and SEM. The attached cells spread on the substrates and expressed important adhesion markers like vinculin and FAK, which are for example responsible for cell survival^51^.

Since we had no access to primary isolated human EPCs, a limitation of the study is that not exactly the same type of progenitor cell for mouse and human studies was used (EPCs versus ECFCs) and therefore we cannot directly compare between species. Nevertheless, since we detected impressive effects of endothelial cell recruitment by DCN and SDF-1α, a combination of these two molecules could be also favorable in order to attract cells after implantation and thus improve material integration and regeneration. Our results indicate that both, DCN and SDF-1α are suitable candidates to generate functionalized off-the shelf implants with *in vivo* colonialization potential.

## Methods

All human and animal cells used in this study were obtained by the sources stated in the relevant sections. All experiments were performed in accordance with the institutional guidelines and regulations of the University of Tübingen, the Fraunhofer IGB Stuttgart, and the Charité Universitätsmedizin Berlin.

### Recombinant DCN production

Commercially available vectors were used for the construction of the DCN production plasmid (Supplementary Fig. S1). The main backbone was created using the pcDNA™3.1 vector. The CMV promotor for DCN expression was used from the pCMV-Tet3G vector, and controlling of dihydrofolate reductase (DHFR) expression was performed by the TK promoter from the pGL4.74(hRluc/TK) vector. A human DCN codon-optimized sequence (GenBank accession number: BT019800.1, 1080 bp) provided with a C-terminus histidine/asparagine tag was synthesized from GeneArt (Life Technologies, USA). To support adherent growth of CHO DHFR^−^ cells (Leibniz-Institut DSMZ-Deutsche Sammlung von Mikroorganismen und Zellkulturen GmbH; DSMZ no.: ACC 126), cells were cultured and maintained in minimum essential medium (MEM) α without nucleosides (22561-021, Life Technologies), supplemented with 10% fetal bovine serum (FBS; 10270106, Gibco, Life Technologies) and hypoxanthine and thymidine (11067-030, Gibco, Life Technologies). Cells were split every 2 to 4 days using 0.25% trypsin-EDTA (1x) with phenol red (25200-072, Life Technologies). For human DCN production, CHO DHFR^−^ cells were transfected with the DCN production plasmid and cultured for two weeks in MEM α, supplemented with 10% dialyzed FBS. Subsequently, single cell cloning was performed and DCN concentrations were determined employing an enzyme-linked immunosorbent assay (ELISA) system (DY143, R&D Systems, USA). With increasing levels of methotrexate (MTX), clones for genomic amplification were selected. To monitor DCN gene and protein levels during MTX selection, ELISA, q-RT-PCR and q-PCR were performed. Clones with the highest level of DCN production were adapted to suspension growth in Erlenmeyer flasks using a serum-reduced medium (DMEM/Ham’s F12 basal medium, 1% FBS dialyzed, 2.5 µM MTX, 2 mM L-Glutamine, 1% penicillin-streptomycin (PS)) and an incubation shaker (Minitron, Infors GmbH, Switzerland). Suspension cell culture conditions were 37 °C and 5% CO_2_ and 85 rotations per minute (rpm). Every 4 to 5 days, DCN-containing medium was harvested and stored at −20 °C before DCN purification.

### DCN purification

The IMAC purification of human DCN tagged with histidine/asparagine was performed with a HisPrep FF 16/10 affinity chromatography column (GE Healthcare, USA), controlled by the FPLC system Äkta Explorer 10 (GE Healthcare). DCN elution was identified by an increased absorption at 280 nm and 256 nm. All DCN samples were pooled and desalted with a HiPrep™ 26/10 desalting column (GE Healthcare), controlled by the Äkta Purifier 100 (GE Healthcare). After washing and concentrating with ultrafiltration units (Vivaspin 20, Sartorius, Germany), the DCN samples were sterile filtered (SCGP00525, Millipore, USA) and subsequently stored at −80 °C. All DCN samples were tested for endotoxin contamination by the Pierce LAL chromogenic endotoxin quantification kit (Thermo Scientific GmbH, Schwerte, Germany) with E. coli LPS as a standard. The level for 50 µg/mL DCN was 1.2 ± 0.31 EU/mL.

### SDS-PAGE and Western blot

DCN samples were mixed with 4x Roti-Load (Carl Roth, Germany) and denatured at 90 °C for 5 min for SDS-PAGE. The samples were run on a NuPAGE®Novex 4–12% Bis-Tris 1.0 mm 12 well gel (NP0322BOX, Life Technologies, USA). For band identification, SeeBlue® Plus2 Pre-Stained Standard (LC5925, Life Technologies) was used. Using an electrical field (30 V, 60 min) in the XCell II™ Blot Module (Life Technologies), the proteins were blotted to a nitrocellulose membrane (Whatman, UK). Proteins were visualized by incubating the membrane for 5 min in a Ponceau-Red solution (Sigma Aldrich, USA). After imaging, the membrane was discolored in a 0.1 M NaOH solution for further specific protein detection. The membrane was blocked with 5% skim milk powder (Sigma-Aldrich) in TBS-T and then incubated with a DCN antibody (1:2000; GTX 101250, Genetex, USA) overnight at 4 °C. After washing with TBS-T, the membrane was incubated with the secondary goat-anti-rabbit IgG H&L (HRP) antibody (1:4000 in TBS-T with 5% skim milk powder; ab6721, Abcam, UK). After 1 h incubation at RT, the membrane was washed and a SuperSignal West Dura Extended Duration Substrate (Thermo Scientific, USA), was applied onto the membrane and the chemiluminescence was imaged using the Luminescent Image Analyzer LAS-1000 plus (FujiFilm, Japan).

### DCN deglycosylation to prove availability of the GAG chain

Deglycosylation of purified DCN was performed with an enzyme mix (P6039S, New England Biolabs, USA) according to the manufacturer’s manual. In addition to the 4 h incubation time described by the manufacturer, an identical reaction mix was incubated for another 20 hours. A non-enzyme-treated sample served as control. DCN deglycosylation was analyzed with SDS-PAGE.

### Co-Immunoprecipitation (Co-IP)

Human recombinant DCN (0.5 µg) and human TGF-β1 from platelets (0.225 µg; T1654-1UG, Sigma Aldrich) were diluted in 500 µL washing buffer (0.1 M NaCI, 0.05 M Tris/HCl pH 7.4 containing 0.04% Tween-20 and 1% BSA). The following agitation was performed on a rotisserie mixer overnight. For Co-IP, 10 µg of the DCN antibody (GTX 101250, Genetex, USA) was incubated with the protein mix for 24 hours. No primary antibody was added to the negative control. The protein complexes with and without the DCN antibody were mixed with protein A magnetic beads (LSKMAGA02, Millipore) on the rotisserie mixer for 2.5 hours at 4 °C, and another 30 min at RT. Using a magnetic stand (LSKMAGS08, Millipore), the magnetic beads were removed and the protein complexes were eluted from the beads. In a next step, the protein complexes were denatured in 30 µL of 1x Roti-Load (K929.2, Carl Roth, Germany) at 90 °C for 10 min. Specific protein bands were detected after SDS-PAGE and Western blot as described before.

### Isolation of human immune cells

After obtaining informed consent and approval by the local Ethical Committee (Charité Universitätsmedizin Berlin; IRB# EA 1/226/14), buffy coats were acquired from the German Red Cross (Berlin, Germany), and peripheral blood mononuclear cells (PBMCs) were isolated and separated by centrifugation for 30 min (800xg, room temperature, no brake) on a Biocoll (Biochrom AG) gradient. Isolated PBMCs were then washed three times with PBS, and were used either for the proliferation assay or for the isolation of monocytes. CD14^+^ monocytes were magnetically-sorted using CD14 MicroBeads (Miltenyi Biotec GmbH, Bergisch Gladbach, Germany) according to the manufacturer’s instructions with a purity of 95–98%. For performing migration assays, polymorphonuclear cells (PMNs) were isolated from peripheral blood from healthy volunteers after informed consent (ethical approval: EA2/139/10 and EA1/226/14). Separation was performed by layering the diluted blood on Polymorphprep (Axis Shield, Liverpool, United Kingdom) followed by centrifugation for 35 min (500xg, room temperature, no brake). PBMCs and PMNs were harvested separately and washed with PBS. The purity of PMNs was determined by flow cytometric analysis as 60–70%. Monocytes were separated from PBMCs as stated before.

### Treatment of innate immune cells with DCN

Monocytes (1 × 10^6^/well) and M0 type macrophages (7 × 10^5^/well) were seeded in 24-well culture plates (Corning B.V. Life Sciences, Amsterdam, The Netherlands) followed by stimulation with 50 µg/mL DCN in complete VLE-RPMI medium (Biochrom AG) containing 10% human AB-Serum (Sigma), 1% PS (Life Technologies) and 1% glutamine (Invitrogen, Carlsbad, USA) and cultured at 37 °C in a 5% CO_2_ incubator for 7 days or 24 hours. Control settings included M0 type macrophages generated by culturing monocytes (2 × 10^6^/mL) for 7 days in 6-well culture plates (Corning B.V. Life Sciences) with 50 ng/mL M-CSF (Miltenyi Biotec GmbH) in complete VLE-RPMI. In parallel, M0 type macrophages were polarized for 24 hours towards becoming M1 type macrophages by adding 20 ng/mL IFN-γ (Miltenyi Biotec GmbH) and 100 ng/mL LPS from *E. coli* O127:B4 (Sigma) or into M2 type macrophages by adding 20 ng/mL IL-4 (Miltenyi Biotec GmbH).

Supernatants from all cultures were collected after 24 hours (macrophages) or 7 days (monocytes) for cytokine detection. Cells were then harvested using 1% (v/v) trypsin/EDTA (Life Technologies) and stained with the following antibodies: CD163-FITC, CD80-PE, HLA-DR-PE/Cy7, CD206-APC (all BioLegend, Fell, Germany), CD14-APC/Cy7 (BD Biosciences, Heidelberg, Germany) and a live/dead marker (Invitrogen, Darmstadt, Germany). Measurements were performed with a FACS Canto II (BD, San Jose, USA) and analyzed using FlowJo Version 8.8.6 software (TreeStar Inc., Ashland, USA). Surface marker expression levels were normalized to the untreated controls, which were set at 1.

### T cell proliferation assay

The influence of DCN on T cell proliferation was analyzed with a CFSE-based assay as described before^52^. Briefly, 96-well culture plates (Corning B.V. Life Sciences) were coated overnight with 0.05 µg/mL anti-CD3 antibody (OKT3; Janssen-Cillag, Neuss, Germany) at 4 °C. After three washes with PBS (Biochrom AG), wells were coated with DCN (50 µg/mL, 6 h, room temperature) followed by an additional washing step with PBS. Then 5,6-CFDA-SE (2.5 µM) labeled PBMCs were seeded at a cell density of 3 × 10^5^ cells/well in complete VLE-RPMI in wells coated with anti-CD3 and DCN, anti-CD3 alone (positive control) or untreated wells (negative control). After 5 days of culture at 37 °C and 5% CO_2_, 100 µL of supernatant were collected for cytokine detection by ELISA. PBMCs were harvested and labeled with the following antibodies: CD8-PE, CD4-APC (both Miltenyi Biotec), CD3-APC/Cy7 (all BioLegend) and a live/dead marker (Invitrogen). Additionally, samples were measured on a FACS Canto II and data were analyzed using FlowJo with normalization to the anti-CD3 control level, set as 1.

### Cytokine detection assays

Supernatants from PBMC proliferation assays were analyzed for IFNγ, TNFα and IL-10 and those from monocyte and macrophage cultures analyzed for TNFα, IL-6 and IL-10 using ELISA kits (BioLegend GmbH) according to the manufacturer’s instructions. Samples were measured on a Micro-plate reader (Bio-Rad, München, Germany).

### Immune cell migration assay

Chemotaxis of monocytes and PMNs was analyzed with a specific 96-well cell migration system (Neuro Probe, Gaithersburg, USA). Briefly, DCN (5, 10, 50 and 100 µg/mL) was added as a chemotactic stimulus in a volume of 37.5 µL per well in diet medium consisting of VLE-RPMI (Biochrom AG) and 0.1% autologous serum. A membrane filter (pore size 5 µm) was placed into the plate and 3 × 10^4^ cells in 40 µL diet medium were added onto the membrane. A diet medium in the well served as a negative control, whereby no concentration gradient was present and only undirected, random cell migration occurred. A stimulus of 10% autologous serum served as a positive control. After 3 hours of incubation at 37 °C and 5% CO_2_, the membrane was carefully removed. Monocytes that adhered to the membrane were fixed with methanol (Merck, Darmstadt, Germany) and labeled with Hemacolor® staining kit (Merck, Darmstadt, Germany). After microscopic documentation using ProgRes® CapturePro 2.8.8 (Jenoptik, Jena, Germany), the number of migrated monocytes was determined using ImageJ Version 1.4.3.67 software (National Institutes of Health, Bethesda, USA). Numbers of migrated PMNs were counted, and the proportion of migrated cells was normalized to the negative control.

### Electrospun scaffold fabrication

The scaffold was electrospun as described before^10^. Briefly, a polymer solution containing 0.08 g/mL poly (ethylene glycol) dimethacrylate (PEGdma; Mn 2,000, 687529, Sigma), 0.08 g/mL poly (L-lactide) (PLA; Mn 59,000, 93578, Sigma), 0.01 g/mL photoinitiator (2-hydroxy-4′-(2-hydroxyethoxy)-2-methylpropiophenone; 410896, Sigma) and 1,1,1,3,3,3-hexafluoro-2-propanol (804515, Merck) was prepared and electrospun with a distance of 18 cm employing a 20 G nozzle and 18 kV^10^. The 75–100 µm thick scaffolds were further UV-irradiated (365 nm) to enable fiber crosslinking. Prior to functionalization, the PEGdma-PLA scaffolds were sterilized using 70% ethanol.

### Scaffold functionalization

Sterilized electrospun PEGdma-PLA scaffolds were either coated with 0.6 µg/mL human SDF-1α (300-28 A, Peprotech, Rocky Hill, US) or 100 µg/mL of the generated human DCN. To enable protein adsorption, scaffolds were incubated for 4 hours at 37 °C with the protein solutions. Unbound protein was removed by washing the functionalized scaffolds with sterile 1x PBS.

### Progenitor cell culture

T17b murine endothelial progenitor cells (mEPCs) were kindly provided by Dr. A.K. Hatzopoulos (Department of Cell and Developmental Biology, Vanderbilt University, USA)^45^. The cells were cultured in Dulbecco’s Modified Eagle Medium (DMEM) supplemented with 20% fetal calf serum (FCS; 10270, Life Technologies, Paisley, UK), 100 U/mL penicillin, 100 µg/mL streptomycin (15140, Life Technologies), 1% non-essential amino acids (11140, Life Technologies) and 0.1 mM b-Mercaptoethanol (28625, Serva, Heidelberg, Germany)^18^. Human endothelial colony forming cells (hECFCs; 00189423, purchased from Lonza, Basel, Switzerland) were cultured in Endothelial Cell Growth Medium (ECGM; C-22020, Promocell, Heidelberg, Germany) containing supplement mix (C-39225, Promocell), 10% FCS, 100 U/mL penicillin, 100 mg/mL streptomycin and 1% Glutamin (25030, Life Technologies). For cell expansion, mEPCs were cultured in suspension, whereas hECFCs were grown adherent on cell culture-treated substrates.

### Cell capturing experiment

SDF-1α- and DCN-coated and uncoated PEGdma-PLA scaffolds (diameter: 8 mm) were placed into wells of a 24-well-plate. 0.5 mL cell suspension of either mEPCs or hECFCs was added to each well, followed by a dynamic incubation of the well-plate under cell culture conditions on an orbital shaker (BellyButton^TM^). This experimental set-up enables a constant flow of cells over the electrospun scaffolds. 5 × 10^4^ mEPCs were pipetted per well, followed by a culture period of 48 hours. hECFCs were cultured for 24 hours. 1 × 10^5^ hECFCs/well were used.

### Detection of attached cells

After the cell-capture experiments, the scaffolds were washed with PBS and fixed with 4% paraformaldehyde. To enable cell counting on the scaffolds, the cell nucleus was stained with 4′,6-Diamidino-2-phenylindoldihydrochlorid (DAPI). Both sides of the scaffold were systematically investigated by taking five images per side (equals ten images per sample) with a Zeiss fluorescence microscope (ObserverZ1, Carl Zeiss GmbH, Jena, Germany). For each condition, eight experiments were performed (n = 8).

### Scanning electron microscopy (SEM)

In order to visualize the morphology of the attached cells, a scanning electron microscope (1530 VP Zeiss, Carl Zeiss GmbH) was used. The samples were fixed with 2% glutaraldehyde and dehydrated with ascending alcohol. After complete drying at room temperature, the cell-seeded scaffolds were mounted onto stubs and sputtered with platinum under vacuum.

### Immunofluorescence staining

The protein-coated scaffolds were stained as previously described^7^. Anti-decorin (1:250; GTX101250, GeneTex Irvine, USA) and anti-SDF-1α (1:200; 500-P87A, Peprotech) served as primary antibodies. The protein was visualized with a fluorescence-conjugated secondary antibody (1:250; A11034, Alexa Fluor® 488 anti-rabbit-IgG, Molecular Probes, Darmstadt, Germany).

After cell culture experiments, the cell-seeded scaffolds were rinsed with PBS and fixed with 4% paraformaldehyde. After blocking unspecific binding sites with a goat serum, the cell-seeded scaffolds were processed as described before^10^. Primary antibodies, including anti-FAK (1:100; ab4803, Abcam, Cambridge, UK), anti-vinculin (1:500; MAB3574, Millipore, Darmstadt, Germany), anti-vWF (1:200; A0082, Dako, Hamburg, Germany), anti-CXCR4 (1:200; ab2074, Abcam) and anti-VEGFR2 (1:100; ab2349, Abcam) were incubated over night at 4 °C. The primary antibody solutions were then removed, followed by multiple washing with PBS, and subsequent incubation with the secondary antibodies (1:250; A-11034 and A-21121, Alexa Fluor® 488, Molecular Probes), together with Alexa Fluor® 546-conjugated phalloidin (1:100; A22283 Alexa Fluor® 546, Molecular Probes). After a 45-minute incubation in the dark, the samples were rinsed with PBS and the cell nuclei were stained with DAPI. Images were obtained by using a Fluorescence Microscope (ObserverZ1, Carl Zeiss GmbH).

### Imaging Flow Cytometry

Cell viability was analyzed using an imaging flow cytometer (ImageStreamX Mark II, Amnis, Seattle, USA). After the cell capture experiment, the cells were removed from the scaffolds applying trypsin for three minutes. The cells from each sample were split into two groups and each of them was washed and finally re-suspended in 50 µL cold FACS buffer (PBS with 2% (v/v) FCS). One served as a control to adjust the system and to exclude autofluorescence, the other one was further processed for staining. In order to label dead cells, we added 5 µL 7-aminoactinomycin D (7-AAD; 559925, BD Biosciences, Franklin Lakes, USA) per 10^6^ cells. Following the incubation of the fluorescent dye for ten minutes on ice, the samples were measured with the same settings as the unstained control. To generate a compensation matrix for later analysis, the stained sample was additionally measured using all emission channels. The software IDEAS® (Merck Millipore, Billerica, USA) enabled further data analysis. In order to obtain adequate cell numbers for imaging flow cytometry, ten scaffolds per experiment were pooled. A total of three experiments for each condition was performed (n = 3).

### Trypan blue viability test

Cell viability was determined by mixing 10 µL cell suspension with 10 µL trypan blue, which labels dead cells in blue. Cell counting was immediately performed under the microscope using a Neubauer chamber.

### Statistical analyses

Unless otherwise noted, data are presented as mean ± standard deviation (SD). For the immune data GraphPad Prism version 6 software (GraphPad Software Inc., La Jolla, USA) was used. Normality of data distribution was evaluated by the D’ Agostino-Pearson omnibus normality test. A one-way ANOVA (RM or Kruskal-Wallis) with Dunnet’s or Dunn’s Post-test was used to compare more than two groups. To compare different cell subsets and different treatments, a two-way-ANOVA with Sidak’s multiple comparison testing was applied. Comparisons between two independent groups were performed with the non-parametric Mann-Whitney U test or the Kolmogorov-Smirnov test for relative values. For cell capture experiments, statistical significance was determined by considering the confidence interval (CI; 95%). In addition, p values were calculated for normal distributed data with a student’s t-test using Excel and Origin software (Origin Pro 8 G, Northhampton, UK). P values less than 0.05 were considered as statistically significant. Outliers were defined by employing the Nalimov test on normal distributed data.

## Electronic supplementary material

Supplementary Figures
