# Supplementary material for: Surface functionalization of electrospun scaffolds using recombinant human decorin attracts circulating endothelial progenitor cells
Source: Sci Rep. 2018 Jan 8;8:110. doi: 10.1038/s41598-017-18382-y (PMC5758628; doi:10.1038/s41598-017-18382-y)
Supplement: Supplementary file 1 — Supplementary Figures [file 41598_2017_18382_MOESM1_ESM.pdf]

Surface functionalization of electrospun scaffolds using recombinant human decorin attracts circulating endothelial progenitor cells

Svenja Hinderer[1,2], Katrin Sudrow[3], Maria Schneider[3], Monika Holeiter[2], Shannon Lee Layland[1,2], Martina Seifert[3], and Katja Schenke-Layland[1,2,4]*

[1] Dept. of Cell and Tissue Engineering, Fraunhofer-Institute for Interfacial Engineering and Biotechnology (IGB), 70569 Stuttgart, Germany

[2] Dept. of Women´s Health, Research Institute for Women's Health, Eberhard-Karls-University Tübingen, 72076 Tübingen, Germany

[3] Institute of Medical Immunology and Berlin-Brandenburg Center for Regenerative Therapies (BCRT), Charité-Universitätsmedizin Berlin, corporate member of Freie Universität Berlin, Humboldt-Universität zu Berlin, and Berlin Institute of Health, 13353 Berlin, Germany

[4] Dept. of Medicine/ Cardiology, Cardiovascular Research Laboratories, David Geffen School of Medicine at UCLA, Los Angeles, CA, USA

Supplementary figures

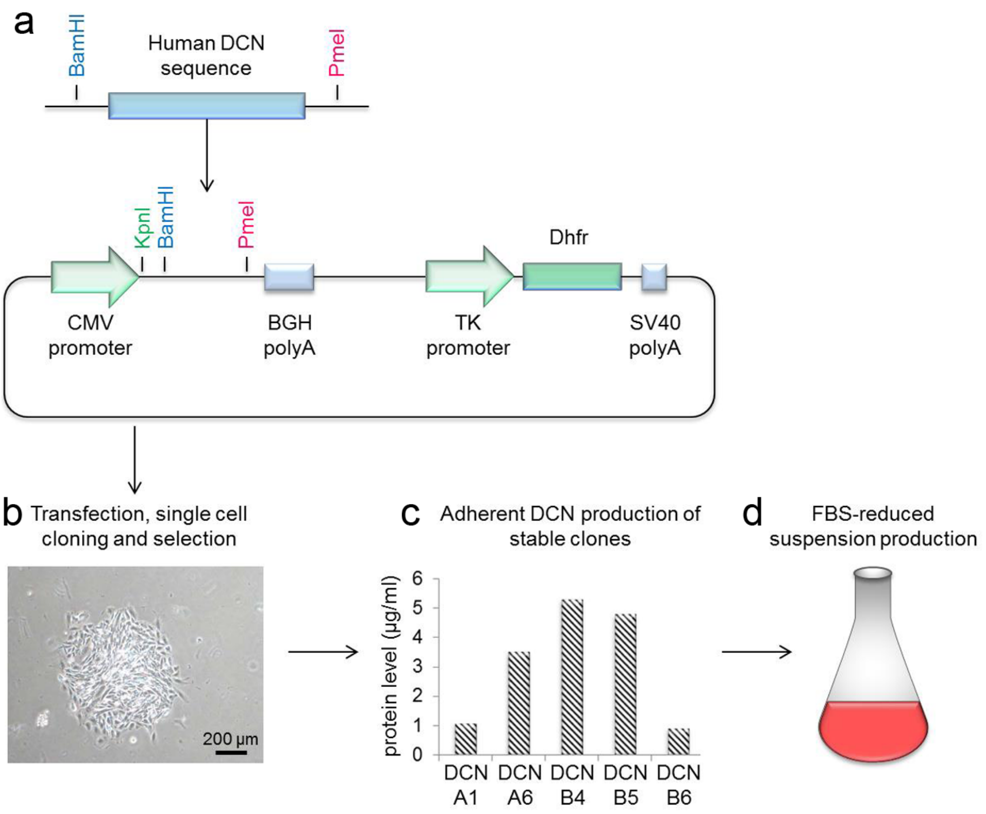

Supplementary Figure S1: (a) Schematic of the production plasmid generation from a synthesized codon-optimized human DCN sequence and the vector containing a wildtype (wt) dihydrofolate reductase (DHFR) expression cassette. **(b)** Adherent CHO cells were transfected with the production plasmid. Following single cell cloning and selection of stable clones for high protein production **(c)**, the clones were adapted to suspension growth with reduced FBS content in Erlenmeyer flasks **(d)**.

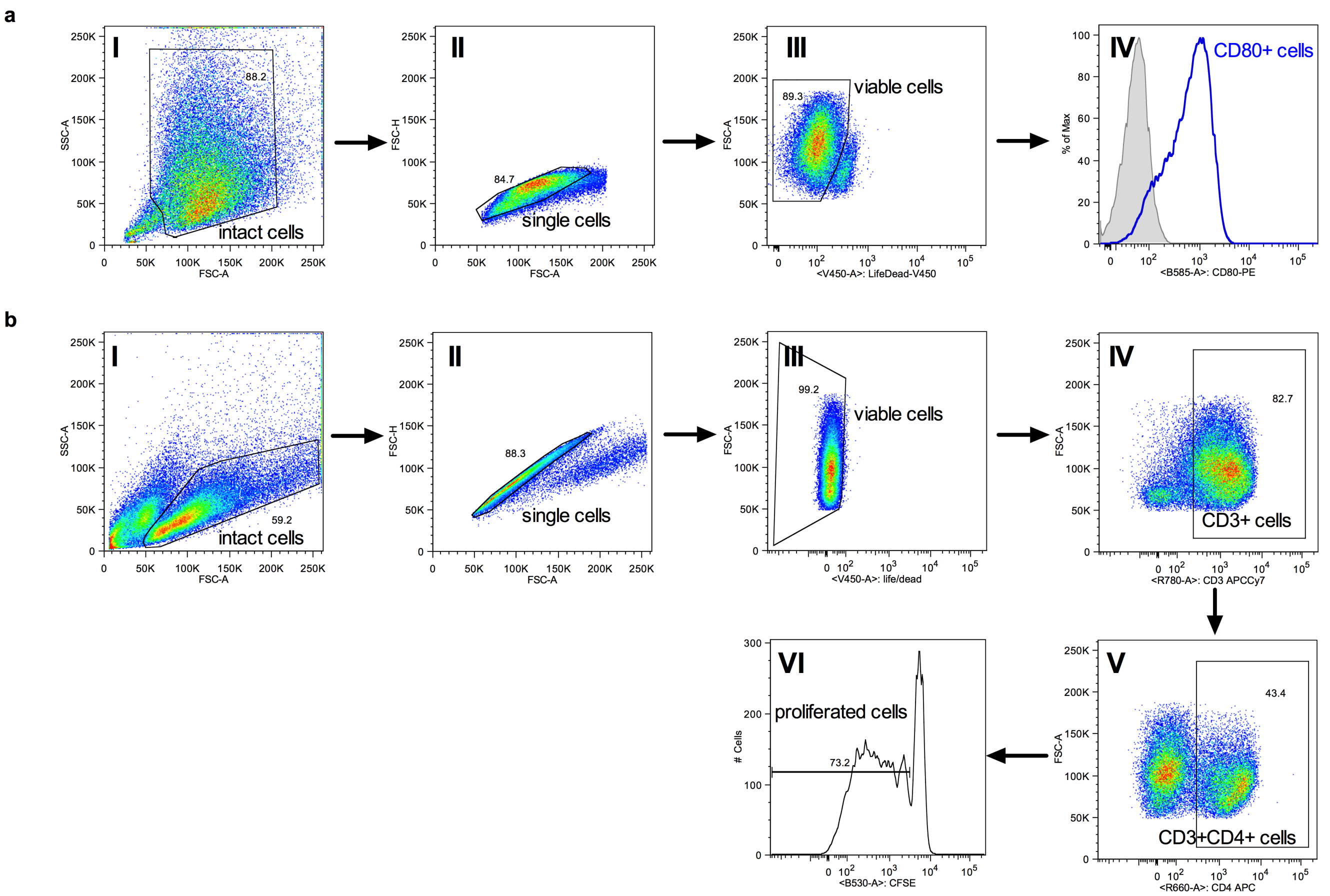

Supplementary Figure S2: The general gating strategies are shown for the analysis of macrophages after 24h-co-culture with DCN **(a),** and for human CFSE-labeled PBMCs after 5 days of co-culture with anti-CD3 and DCN **(b)**. Macrophages were first gated based on their typical location in a scatter plot, **(a, I),** verified as single cells **(a, II)** and viable **(a, III)** before marker-positive cells (here, CD80+ cells) were determined in a histogram **(a, IV).** PBMCs were first gated based on their typical location in a scatter plot, **(b, I),** verified as single cells **(b, II)** and viable **(b, III),** before CD3+ **(b, IV)** and then CD3+CD4+ cells **(b, V)** were determined. Thereafter, the percentages of proliferated cells were analyzed **(b, VI)**.

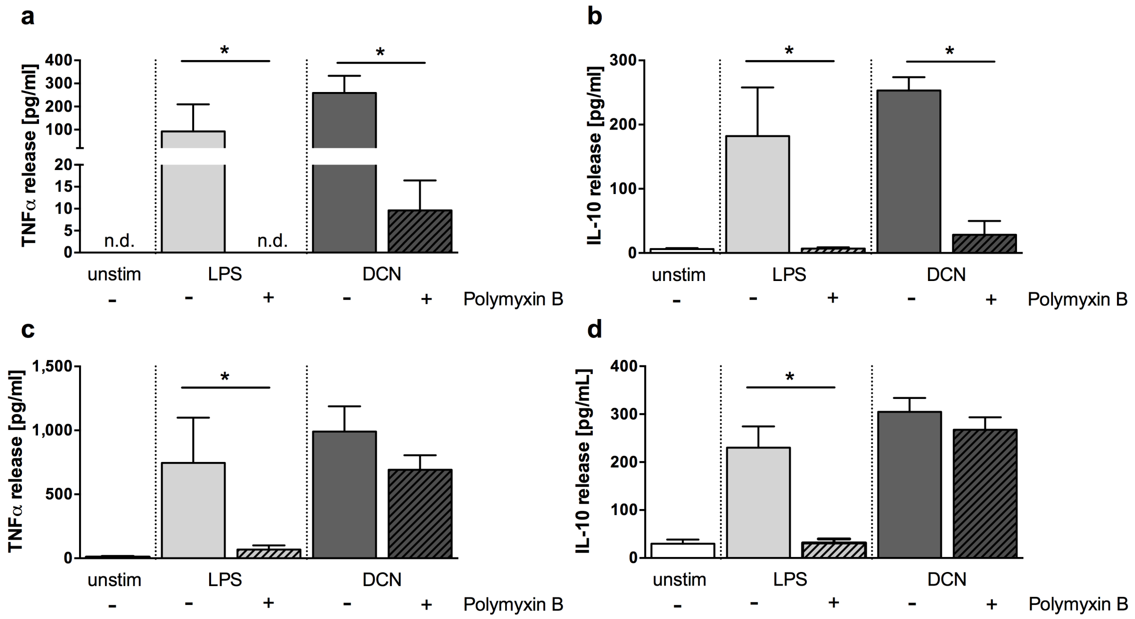

Supplementary Figure S3: Secretion of cytokines in DCN-stimulated monocyte and macrophage cultures. The relative release of TNFα **(a,c)** and IL-10 **(b,d)** after 7 days in co-culture with monocytes **(a,b)**, and 24 hours in co-culture with M0 macrophages (**c,d)** with either DCN (50 µg/mL) or lipopolysaccharide (LPS; 10 µg/mL) alone or after pre-incubation with Polymyxin B (50 µg/mL) is shown as mean ± SD in comparison to unstimulated (unstim) cultures. Data were analyzed by Mann-Whitney U test; * $p<0.05$ between Polymyxin B treated or untreated samples with n=5.

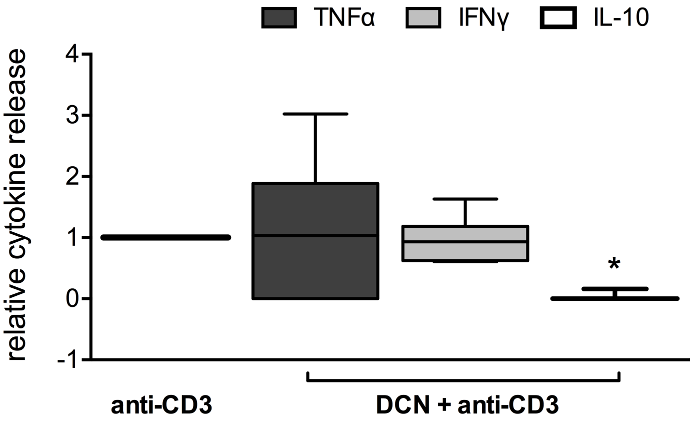

Supplementary Figure S4: The relative cytokine secretion of PBMC co-cultures after 5 days with either anti-CD3 alone or DCN combined with anti-CD3 is depicted for TNFα, IFNγ and IL-10. Data for proliferation and cytokine release were analyzed by one-sample t-test; * $p<0.05$ relative to the anti-CD3 control with n=3-5.

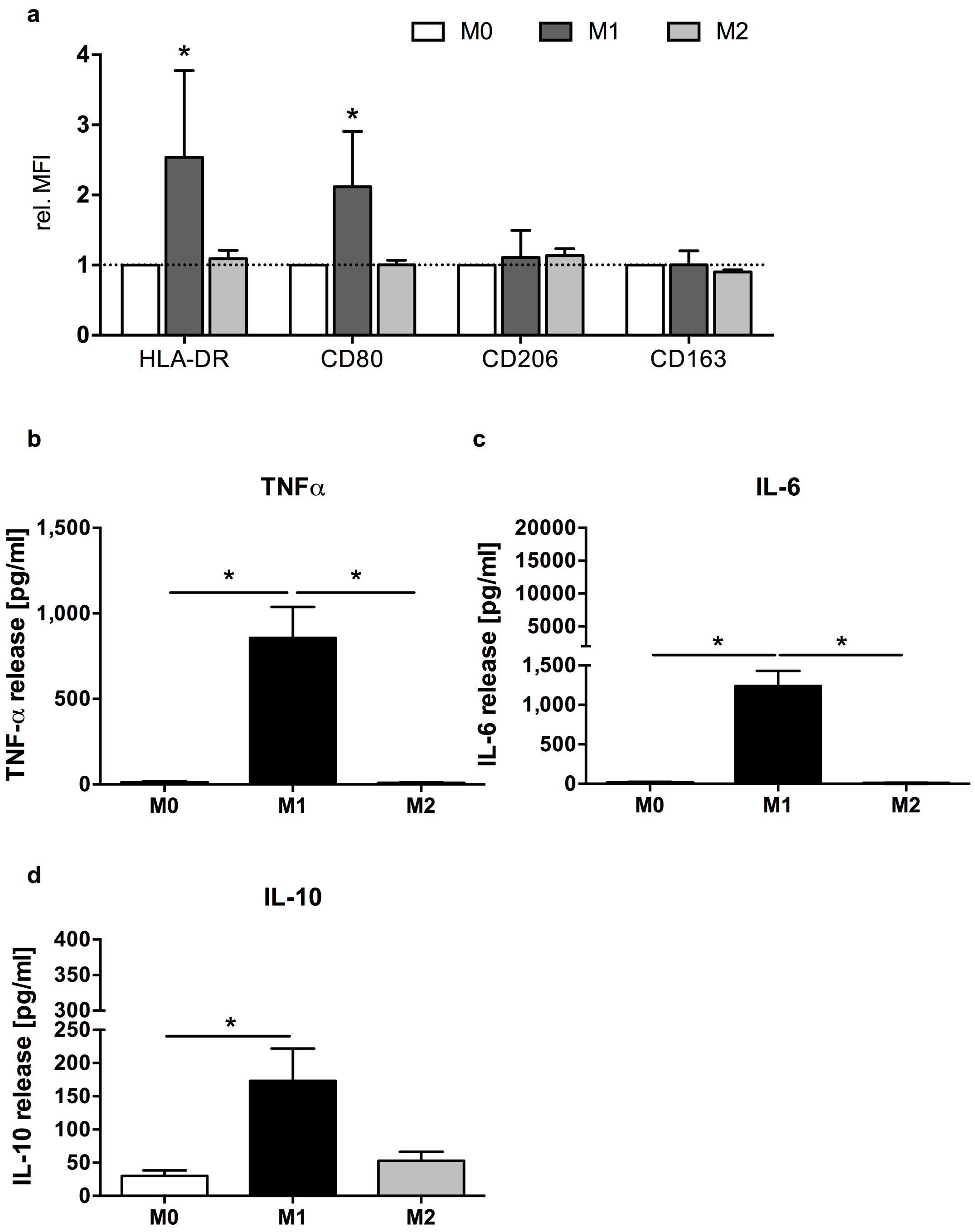

Supplementary Figure S5: Immunological features of polarized human macrophages. A characteristic surface marker expression and cytokine release for M0, M1 and M2 type macrophages is shown. Monocytes were differentiated into M0 type macrophages for 7 days using 50 ng/mL M-CSF. M0 type macrophages were polarized for 24h either towards M1 type macrophages by adding 20 ng/mL IFN-γ and 100 ng/mL LPS, or into M2 type cells by adding 20 ng/mL IL-4. **(a)** Surface marker expression of HLA-DR, CD80, CD206 and CD163 was analyzed by flow cytometry and the relative mean fluorescence intensities (MFI) are shown as mean ± SEM for n=5 independent experiments. Cell culture supernatants were harvested and analyzed for the secretion of **(b)** TNFα, **(c)** IL-6 and **(d)** IL-10 by ELISA. The cytokine release is shown as mean ± SD for n=5 independent experiments; * $p < 0.05$.

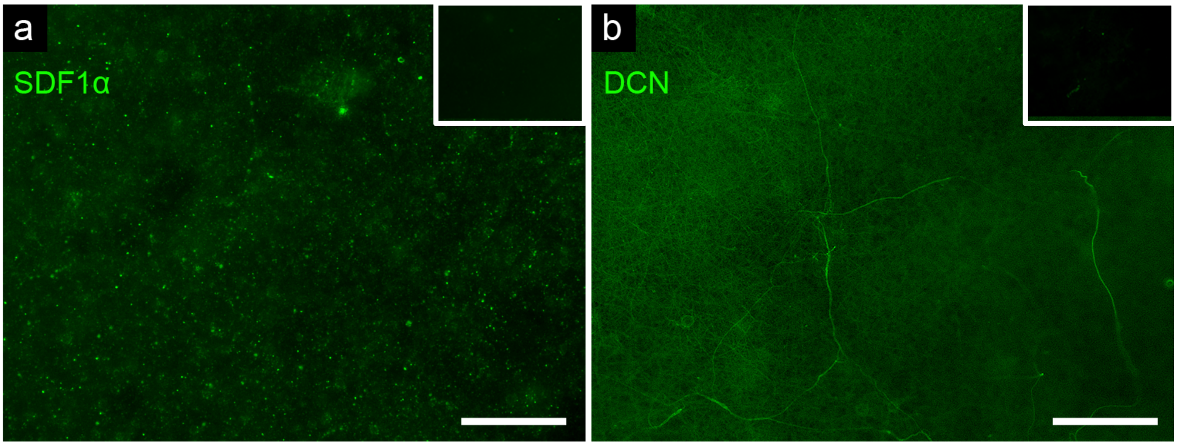

Supplementary Figure S6: Immunofluorescence staining of PEGdma-PLA scaffolds coated with either **(a)** DCN (green) or **(b)** SDF-1α (green). Negative controls are displayed in the white box in the upper right corner of each image. Scale bars equal 100 µm.

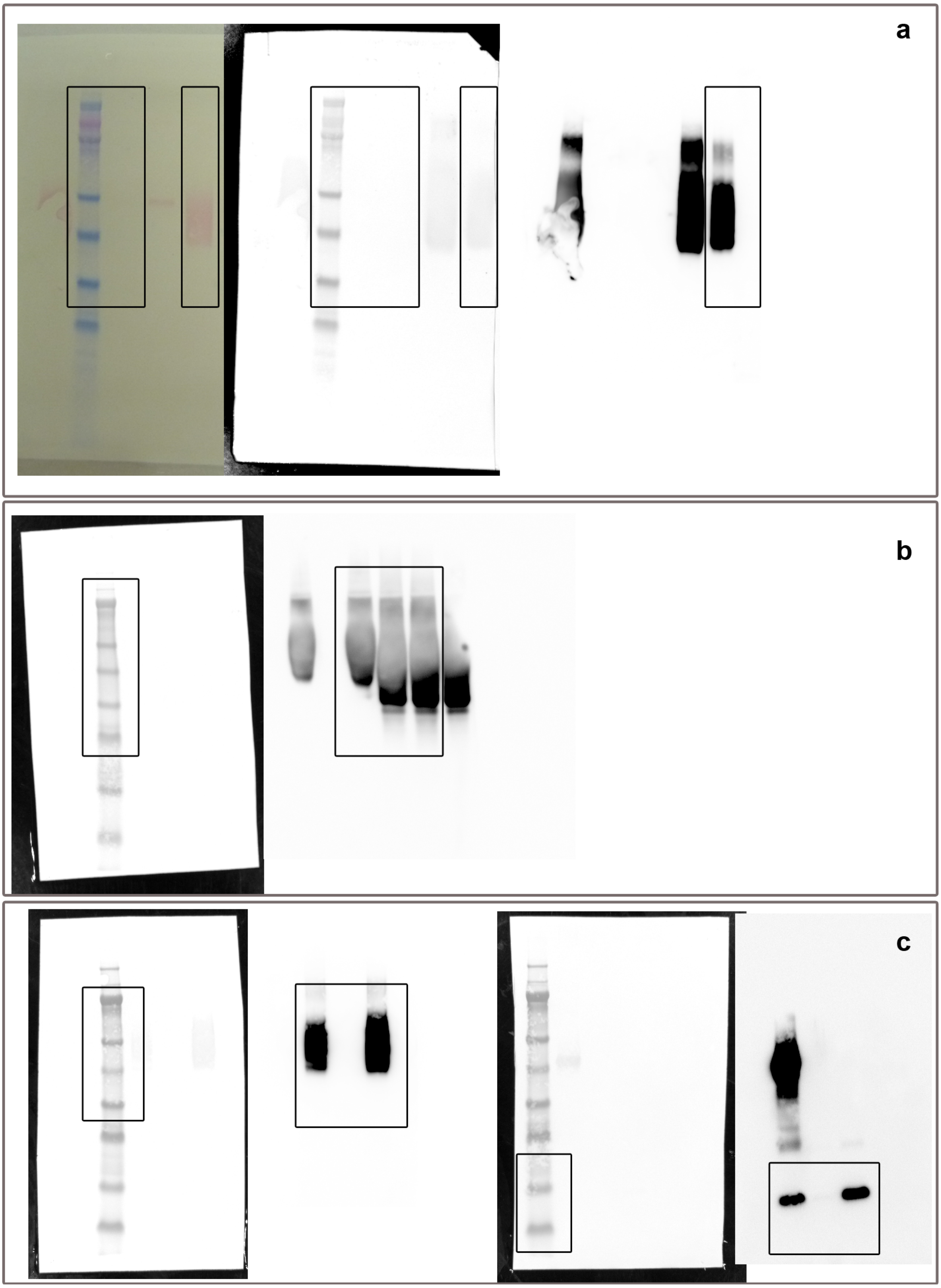

Supplementary Figure S7: Western blot and SDS-PAGE of the full membrane original images. **(a)** Belongs to Figure 1a and b, **(b)** displays original images of Figure 1c and **(c)** shows full image of Figure 1e and f. The black boxes are the selected areas used in Figure 1.
